# Fecal diverting device for the substitution of defunctioning stoma: preliminary clinical study

**DOI:** 10.1007/s00464-018-6389-4

**Published:** 2018-08-14

**Authors:** Jae Hwang Kim, Sohyun Kim, Sang Hun Jung

**Affiliations:** Department of Surgery, College of Medicine, Yeungnam University, 170 Hyeonchung-ro, Nam-gu, Daegu, 42415 South Korea

**Keywords:** Anastomotic leakage, Stoma, Ileostomy, Low anterior resection, Fecal diversion, Fecal diverting device

## Abstract

**Background:**

A novel fecal diverting device (FDD) made for the prevention of sepsis resulting from anastomotic leakage (AL) was tested successfully in an animal study. This study was undertaken to evaluate the clinical safety and effectiveness of the FDD.

**Methods:**

A prospective observation trial was implemented in a tertiary referral university hospital. The study enrolled patients who needed a defunctioning stoma to preserve low-lying rectal anastomosis. The FDD was fixed to the proximal colon 15 cm from the anastomosis and scheduled to divert feces for 3 weeks. The duration could be extended for more than 3 weeks if AL was noted. Postoperative evaluations of AL were performed by obtaining a computed tomography (CT) scan after 1 week and a contrast study after 3 weeks. The outcomes were FDD-related complications, and the capacity of the FDD to preserve the anastomosis. The median follow-up period was 10 (range 5–40) months.

**Results:**

Thirty-one patients, including 5 benign cases, were evaluated. There was no case of stoma conversion or surgical re-intervention. Evidence of AL was identified in 10 (32%) patients using the CT scan at 1 week after surgery. However, in the contrast study at 3 weeks after surgery, only 5 cases of AL sinus were noted. Conservative treatments including 1–3 weeks prolongation of FDD maintenance were enough to preserve the anastomosis. There were 3 cases of partial colonic wall erosions at the FDD attachment area. All of these patients showed improvement with conservative treatment. The limitations were that the study was performed in a single institute and without a control group.

**Conclusions:**

The FDD showed a sufficient capacity of fecal diversion and maintenance duration that prevented aggravation of sepsis in the case of AL without significant complications.

Fecal diversion with stoma is considered to be the best option to prevent the catastrophic cascade after anastomotic leakage (AL) associated with lower rectal resection [1–3]. However, this procedure cannot, by itself, prevent leakage [4]. The morbidity and mortality associated with stoma remain as the important clinical consideration [5]. An impaired quality of life and the economic burden for patients represent other disadvantages associated with stoma [6].

The basic mechanism of defunctioning stoma is diverting feces from the anastomotic wound. By doing this, inflammation at the anastomotic wound can be decreased due to a reduction of infection sources. Several tube-structured devices have been attempted to replace the temporary stoma [7–11]; however, they have not been successful. Their limitations, such as limited maintenance duration, incontinence, and questionable safety, may be the main cause of this failure. Anastomotic wound healing usually occurs in a week [12]; however, some conditions need more time for healing. Delayed wound healing may occur in patients with preoperative chemoradiation therapy, long-term use of steroids, AL, or various other conditions [13–15]. If these factors are present, the time for anastomotic wound healing may be prolonged.

A novel tube device, the fecal diverting device **(**FDD, Yushin Med. Co. Seoul, Korea), was developed recently (Fig. 1). It showed a successful fecal diversion and long maintenance duration of up to 82 days in an animal study [16]. Thus, the FDD could prevent development of serious septicemia in conditions of AL.

**Fig. 1:**
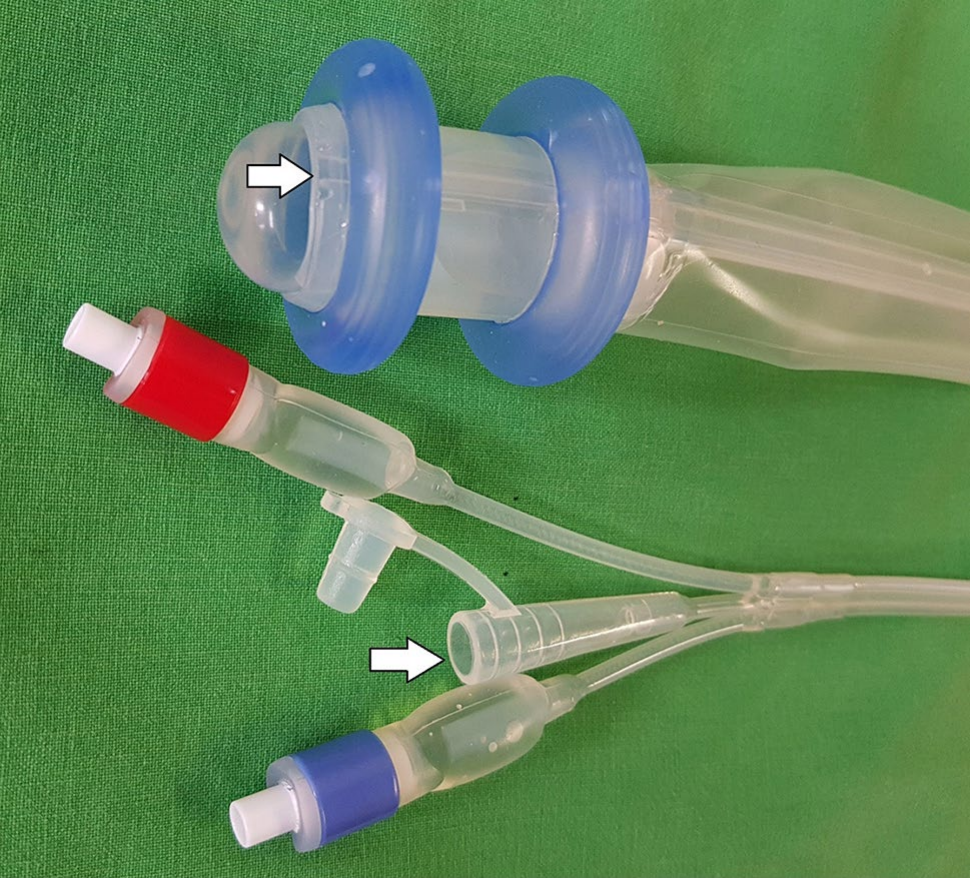
Fecal diverting device (FDD) with inner and outer balloon inflated. The white arrow indicates the channel for irrigation water infusion. The catheter complex tip consists of a blue tip for outer balloon, red tip for inner balloon control, and water infusion port. The catheter complex tip will be fixed to the low abdomen or upper thigh with an adhesive plaster

Before the current study, we performed a pilot study of the FDD in 8 patients who required fecal diversion. We noted that the results were similar to those of the animal study.

In the present clinical study, we attempted to identify the safety and effectiveness of the using the FDD system in patients with low-lying colorectal anastomosis.

## Materials and methods

The study was designed as a prospective observation trial. All the procedures were performed by experienced colorectal surgeons in a tertiary referral university hospital. The study was approved by the ethical review board at Yeungnam University and performed in accordance with the ethical standards of the Declaration of Helsinki.

The participants were patients who needed a defunctioning stoma after low-lying colorectal anastomosis, regardless of whether the cause was benign or malignant. A standard stapled or hand-sewn extraperitoneal anastomosis was created within 8 cm from the anal verge. Patients who were aged < 18 years, had an American Society of Anesthesiologists score > 3, or who were pregnant were excluded. All the patients and caregivers were informed in detail about the procedure, including the risks and benefits, and were invited to participate. Written consent was obtained before surgery or during surgery.

Any mortality and morbidity data of the enrolled patients that were not associated with the FDD were excluded from analysis.

### The FDD system

**Fig. 2:**
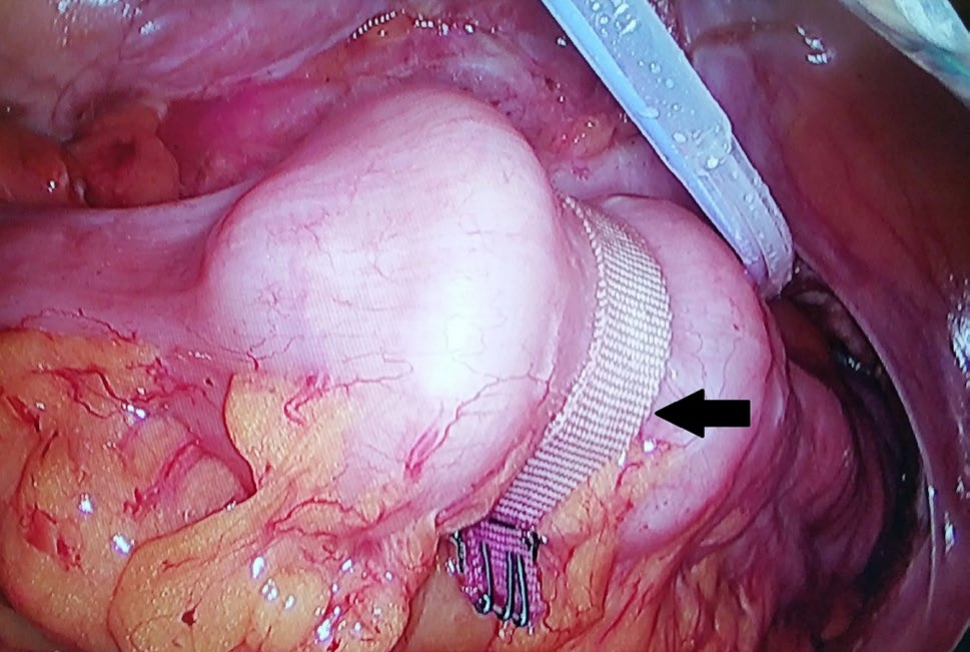
The FDD was attached in the colon by the extraluminal absorbable PGLA mesh band (BAND) indicated by a black arrow

The FDD system consists of 2 main devices: the FDD and a band (BAND) for fixing the FDD as shown in Fig. 2.

**Fig. 3:**
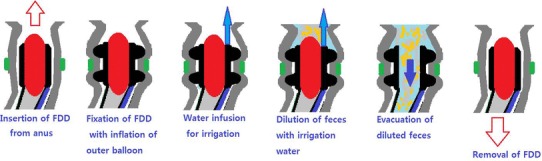
The role of the outer and inner balloons of the fecal diverting device

The FDD is a silicone tube device [16], designed for the protection from fecal contamination of an anastomotic wound using fecal diversion. The FDD is composed of 2 parts, a solid tubular head portion and a thin tubular tail portion. At the head portion, there are 2 outer balloons and 1 inner balloon. The outer balloons are used to attach the FDD at the colon. The inner balloon is used for stop-flow in the tube. A channel for the infusion of irrigation water is located parallel to the catheters for ballooning. The function of the balloons during the FDD procedure is illustrated in Fig. 3. The tail, i.e., the thin tube below the head portion, is long enough to hang out from the anus.

**Fig. 4:**
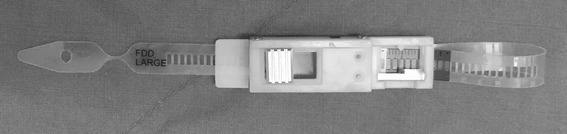
An automatic tension measuring instrument (ATMI, JSR medical Inc, Daegu, Korea). The narrow part of the transparent band breaks automatically if tension reaches a certain point while it is wrapped around the colon. This point indicates a safe compression pressure of the band around the bowel

A BAND is an absorbable poly lactic-co-glycolic acid (PGLA) mesh band (NEOSORB MESH®, Samyang Biopham. Co. Daejeon, Korea) with a half-life of 6 weeks. The extraluminal BAND wraps the waist made between the 2 outer balloons to attach the FDD at a certain location of the bowel (Fig. 2). For the safe application of the BAND, an automatic tension measuring instrument (ATMI, JSR medical Inc, Daegu, Korea) was newly developed and applied in the current study (Fig. 4).

### The FDD procedure

#### Intraoperative FDD installation procedure

**Fig. 5:**
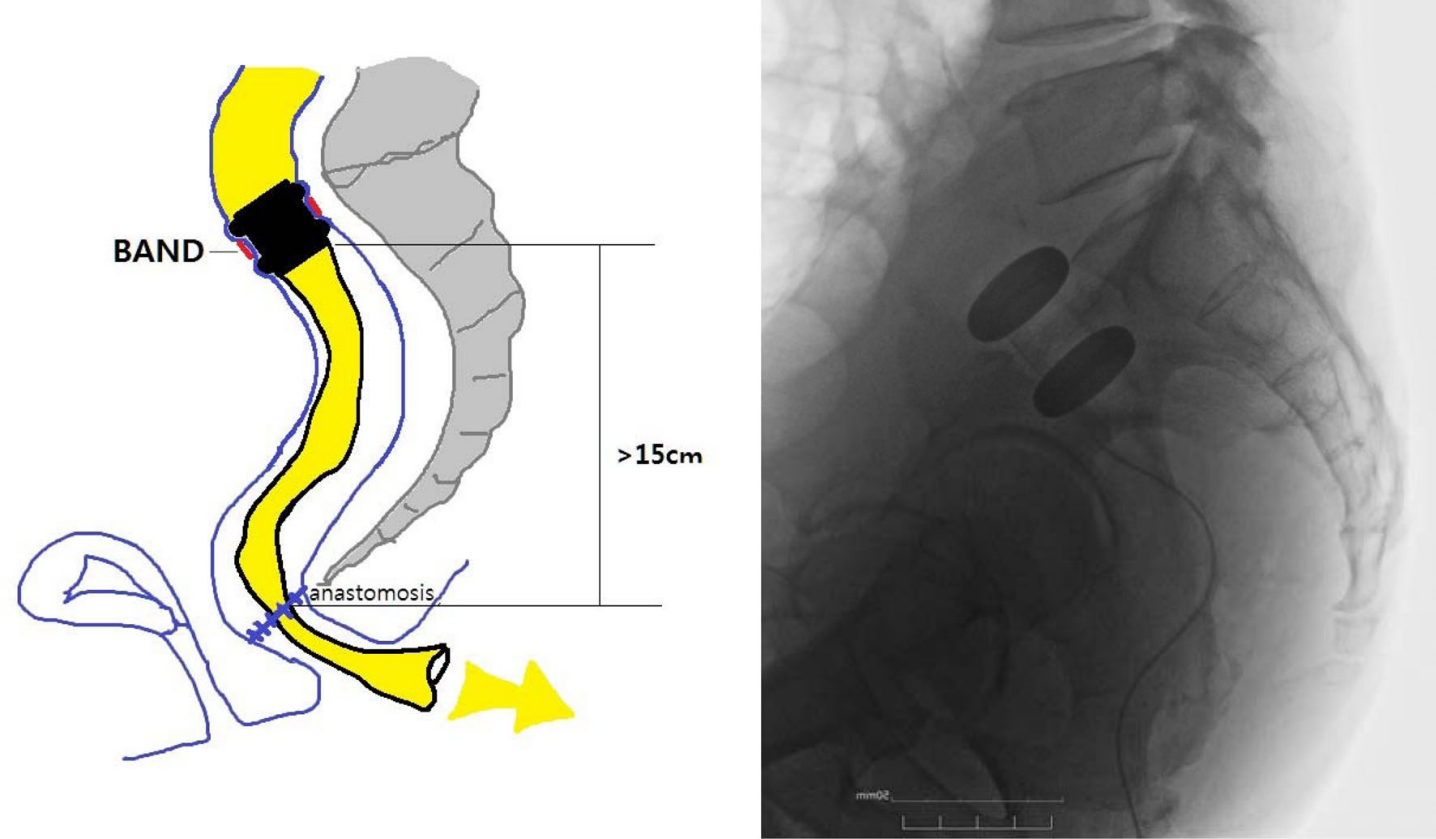
Schematic drawing of fecal diverting device in the colon

Following removal of the pathological specimen, an ATMI was inserted in the mesocolon to measure an adequate length of the BAND. The resulting length was marked on the BAND. The BAND was then introduced and hung at the ATMI-penetrated mesocolic opening with loose suturing using 2 silk sutures (2–0) at the marked point of the BAND. Anvil insertion and circular stapling were performed by a standard stapling technique after setting up the BAND. After anastomosis, an FDD was inserted from the anus to the BAND site with the inner balloon inflated and outer balloons deflated. Two sutures at the marked points of the BAND were tightened after the inflation of the outer balloons (Fig. 5). The procedure was applied in open or laparoscopic conditions with little difference.

#### Postoperative maintenance period

There was no restriction of postoperative oral intake. Daily bowel movement with twice daily irrigation of tepid water via the FDD started a day after intake of a full liquid diet. The patients and caregivers were educated about the mechanism (Fig. 3) of the irrigation procedure and encouraged to perform the procedures.

Patients or guardians were ordered to record the volume of infused irrigation water for the evaluation of adequate irrigation water volume. We recommended to infuse water until the patient felt abdominal discomfort. The volume was counted in 100 mL increments for convenience.

Patients or guardians were also ordered to record the evacuation time. Time was counted in 5-min increments.

The scheduled maintenance duration of the FDD was 3 weeks. If there was AL present, the duration was prolonged. Removal of the FDD was performed with fluoroscopic guidance without anesthesia.

### Postoperative study

One week after surgery, an abdominal computed tomography (CT) scan was scheduled to detect any hidden AL. If there was any evidence of AL clinically before 1 week, a CT scan was performed without delay. The findings were evaluated by experienced radiologists and participating surgeons.

Three weeks after surgery a water-soluble contrast study was performed. If there was no evidence of AL, the FDD was removed. If there was evidence of sinus, the result of AL, the patients were recommended to maintain the FDD for an additional 1–3 weeks. The actual duration was determined by the surgeons. The maximum duration of the FDD maintenance was 6 weeks due to the half-life of the BAND. Sigmoidoscopic examination for the evaluation of the anastomotic site and possible colonic wall injury at the FDD attachment area was performed immediately after the FDD was removed.

#### Follow-up evaluation

The Clavien–Dindo classification (Dindo) was applied for the evaluation of possible surgical complications of the FDD and anastomosis. The definition of AL in this study was a defect in the intestinal wall integrity at the colorectal or coloanal anastomotic site, which led to communication between the intraluminal and extraluminal compartments. A pelvic abscess close to the anastomosis is also considered as AL [17]. Additionally, peri-anastomotic air and peri-anastomotic fluid collection were included [18].

#### Endpoints

The primary purpose of the study was to determine the safety and effectiveness of the FDD system. Safety was assessed by identification of significant intra-abdominal complications caused by bowel wall injury at the FDD attachment area. Inadvertent adverse events caused by FDD installation, maintenance, removal and anodermal injury were also included. Effectiveness was assessed by the capability of complete fecal diversion for a sufficient duration in the case of AL to avoid defunctioning stoma.

The secondary purpose was to identify the adequate volume of irrigation water and adequate evacuation time of diluted feces.

## Results

**Fig. 6:**
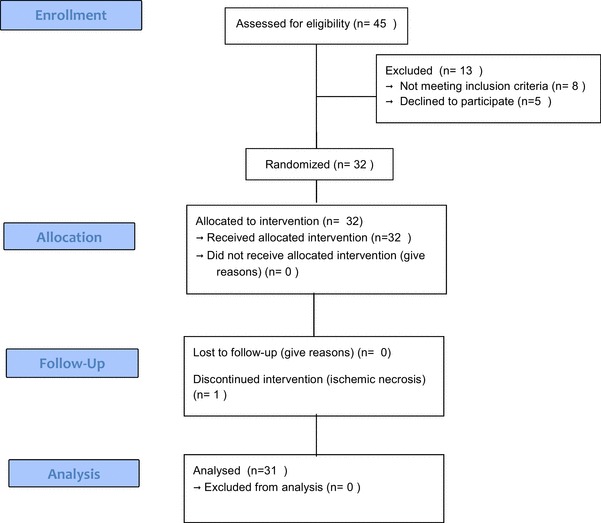
Study flow chart

Thirty-two patients (19 males) with benign or malignant colorectal disease were enrolled between October 2014 and September 2017. One patient was excluded. The remaining 31 cases were included in the analysis for the current study (Fig. 6). The median follow-up period was 10 (range 5–40) months. Baseline clinical characteristics are listed in Table 1.

**Table 1:** Clinical characteristics

Characteristic	All patients (*n* = 31)
Age (year)	70.5 (range 39–86)
Sex (male/female)	19:12
ASA score	
1	10 (32.3)
2	18 (58.1)
3	3 (9.7)
Anastomotic height (cm)	5.9 ± 2.8
Lesion of colon	
Malignancy	26 (83.9)
Benign	5 (16.1)
PCRT	7/26 (26.9)
Maintenance of FDD (day)	23.6 ± 6.1
Hospital stay after operation (day)	13.9 ± 5.8

Values are presented as mean ± standard deviation or number (%)

*ASA score* physical status classification system of American Society of Anesthesiologists, *PCRT* preoperative chemoradiation therapy, *FDD* fecal diverting device

### Exclusion

Permanent ileostomy conversion was performed in 1 patient (female, 78 years) because of a long segment of remaining colonic necrosis. There was no evidence of AL or BAND site abnormality. The patient experienced low anterior resection with high ligation. However, she had a history of right hemicolectomy for ascending colon cancer 12 years prior. The cause of necrosis was ischemia caused by the poor blood supply from the only remaining midcolic artery.

### Endpoints

#### Safety of FDD

The time of FDD removal for all participating patients was decided by the surgeons. The average duration of FDD use was 23.6 ± 6.1 days. The average duration of hospital stay was 13.9 ± 5.8 days. There were 3 partial colonic wall erosions (3/31, 9.7%) noted at the BAND area on days 22, 26, and 36 after surgery. Detection was possible by using a water-soluble contrast study and sigmoidoscopy. Two cases were asymptomatic, and no other treatment was needed (Dindo Grade I). For the other patient, the erosion caused a pericolic abscess; however, it improved with antibiotic treatment (Dindo Grade II).

The follow-up of these patients was uneventful, and there was no evidence of wall defect or stricture in follow-up colonoscopic examination up to 9 months afterward.

Intraoperative installation of the FDD was composed of an intraoperative BAND application and FDD insertion via the anus. There was no failure of the insertion procedure and no traumatic disruption of the anastomotic wound.

During the maintenance period, all patients experienced twice daily irrigation with the FDD for their bowel control. The majority of the irrigation procedures were performed by the patients themselves without significant difficulty. If it was difficult for elderly or handicapped patients to perform the procedure by themselves, healthy caregivers were able to help them easily.

There was no failure of FDD removal and removal of the FDDs was uneventful. In a few instances, there was mild resistance when the solid portion of FDD was passing the anastomotic area. However, it was overcome by slow and gentle pulling of the device.

There was no significant anodermal or perianal skin problem that required medication or treatment.

#### Effectiveness of the FDD in case of AL

Ten cases (32%) of AL were detected by the CT scan examination at 1-week postoperative evaluation. Eight (33%) and 2 (29%) ALs were noted in patients without (*n* = 24) and with (*n* = 7) preoperative chemoradiation, respectively. These were classified as Dindo Grade I (3), Grade II (6), and Grade IIIa (1) surgical complications. The Grade III complication was noted in a patient with local sepsis because of a large peri-anastomotic fluid collection on the 4th postoperative day. Septic symptoms improved dramatically with ultrasonogram guided percutaneous drainage. There were no patients with stoma conversion due to the failure of fecal diversion with the FDD. The contrast study after 3 weeks revealed 5 (16%) cases of remaining sinus in the previous 10 AL cases. Prolonged FDD maintenance was decided in these 5 cases and resulted in a median of 36 days (range 27–43 days) of maintenance duration. The final contrast study before the removal of the FDD revealed a persistent chronic sinus in 4 (13%) cases. No additional defunctioning stoma was provided to these patients.

In the follow-up, after more than 6 months and up to 18 months, there was no recurrence of an anastomotic wound problem in the patients with chronic sinus.

### Secondary endpoints

The median volume of tepid irrigation water was 800 (range 400–1100) mL. The median evacuation time was 20 (range 15–35) minutes. After evacuation, only minimal soilage occurred before the next irrigation.

## Discussion

### The advantage of FDD

The FDD in the current study is a kind of bypass tube device. The FDD, unlike previously introduced devices, overcame most of the existing limitations, such as insufficient and uncontrollable maintenance duration, and incontinence. The maintenance duration of FDD can be controlled by surgeons and can be prolonged for up to 6 weeks. A twice daily irrigation procedure can help most patients to achieve continence.

### Safety

The most serious anticipated adverse effect in this FDD concept is bowel injury, such as necrotic perforation or erosion due to colonic wall ischemia. We were able to estimate the safe pressure range from the previous animal study [16] and could make a device ATMI. This worked well and resulted in no loss of device and no acute intra-abdominal surgical complication.

There was 1 case of pericolic abscess at the erosion site, meaning erosion perforation of the colonic wall. We suppose that the cause of local sepsis might delay wound healing between irradiated adjacent structures [13]. The other 2 patients with erosion required no admission and no additional medication. The sigmoidoscopic examination revealed colonic edemas above the anastomosis in 1 patient. The second patient with erosion had a history of frequent strong pulling of the FDD catheter complex. The patient fixed the catheters at her abdomen tightly. Even though erosion developed at a rate of 10%, the absence of treatment or conservative treatment was enough to solve the problem. In the previous animal study, there was a 15% erosion rate; however, there were no serious conditions that required re-intervention [16]. Nevertheless, it would be preferable if no erosion developed, even if it did not require a surgical re-intervention. Future improvement of the FDD should lead to increased safety of the device.

### Effectiveness

In the current study protocol, the scheduled maintenance duration was 3 weeks. It is known that the collagen deposition at an anastomotic wound is highest 6–12 days after surgery [12]. However, this mechanism might be disturbed due to various causes and results of delayed wound healing [13–15]. In our current study, maintenance duration of 10 patients with AL ranged from 27 to 43 days, which is 1–3 weeks longer than that of the 21 patients without AL. However, there was no increased incidence of complication.

The AL detection rate in our study is higher at 32% than other reports in the literature. We suggest that the reason might be routine evaluation with a CT scan at 1 week after surgery. This high rate is similar to other studies that included early routine evaluation of AL. Pakkastie et al. reported 39% of AL incidence with a routine contrast study approximately 1 week after surgery in their randomized controlled trial [19]. Tagart reported 36% of AL with routine contrast approximately 2 weeks after surgery [20]. If we include the 4 cases (12.9%) of persistent sinus after 26–43 days of fecal diversion, the incidence rate of AL would be similar to the other results evaluated before stoma closing [21, 22].

In this study, it is notable that there were no complications that required surgical re-intervention even though there was a high rate of AL. We suppose that the capacity of fecal diversion of FDD is comparable to the conventional defunctioning stoma.

During the maintenance period of the FDD, we controlled bowel movement with irrigation twice a day. Fecal contents cannot pass easily through non-peristaltic tube structures without modification of the solid characteristics; therefore, dilution of feces is inevitable to avoid passage disturbance. We targeted dilution before the feces solidified. From the pilot study, we found that once daily dilution sometimes resulted in passage disturbance. The time for the meals to reach the cecum, known as the orocecal transit time, is about 90 min [23], and the normal transit time of the ascending colon is 24 h [24]. Therefore, we elected for twice daily irrigation with tepid water in order to dilute the feces in the ascending colon for easy evacuation.

A limitation of this study is that all procedures were performed by experienced colorectal surgeons from a single institution with experience in animal studies. Additionally, the population of patients with preoperative chemoradiation was relatively small for the analysis of safety and effectiveness of the current procedure. Finally, we did not perform quantitative analysis regarding patient tolerance.

In conclusion, the FDD procedure showed no serious complications associated with the attachment of the FDD that required re-intervention. The capacity of fecal diversion in a situation of anastomotic wound disruption was sufficient to prevent aggravation of septicemia and to avoid re-laparotomy or temporary stoma. The FDD could be maintained for up to 6 weeks without serious complication.
